# Daytime sleepiness and academic engagement among university students: mind wandering as a partial mediator and physical activity as a limited moderator

**DOI:** 10.3389/fpsyg.2026.1854161

**Published:** 2026-06-25

**Authors:** Yanli Tan, Shiqi Liu, Liuhong Zang

**Affiliations:** School of Sports Science, Xinjiang Normal University, Urumqi, China

**Keywords:** academic engagement, daytime sleepiness, mind wandering, moderated mediation, physical activity, university students

## Abstract

This cross-sectional study examined whether mind wandering statistically accounted for the association between daytime sleepiness and academic engagement, and whether physical activity served as a boundary condition in this association. University students in a southern province of China, completed an anonymous online questionnaire. After prespecified quality screening, 1,377 valid responses were included in the main analyses and 1,371 complete cases were included in the moderated mediation analysis. Daytime sleepiness was associated with lower academic engagement and more frequent mind wandering. Mind wandering partially accounted for the association between daytime sleepiness and academic engagement, with an indirect effect of −0.122 (95% bootstrap CI [−0.152, −0.094]), representing approximately 31.8% of the total association. Physical activity showed a statistically detectable interaction with daytime sleepiness in relation to mind wandering (*B* = −0.005, *p* = 0.003), but the incremental explanatory value and the moderated mediation index were very small [index = 0.001; 95% bootstrap CI (0.000, 0.002)]. These findings should be interpreted as cross-sectional associations. Mind wandering appeared to be a relevant cognitive correlate within the proposed model, whereas the practical relevance of the physical activity moderation was limited and should not be interpreted as evidence of a strong buffering effect.

## 1 Introduction

Sleep–wake regulation is important for university students’ daytime alertness and learning functioning. Excessive daytime sleepiness has been associated with poorer well-being and academic attainment in student samples (Howells and Smith, 2019), lower academic performance in medical students (El Hangouche et al., 2018), and broader sleep-related and psychological difficulties (Perotta et al., 2021; Maithani et al., 2024). These findings suggest that daytime sleepiness is not merely a subjective complaint but a marker of impaired daytime functioning that may be relevant to students’ academic lives.

Academic engagement is a central indicator of positive student functioning because it reflects vigor, dedication, and absorption in learning activities rather than achievement alone. The Utrecht Work Engagement Scale for Students has shown support for this multidimensional structure in university samples (Schaufeli et al., 2002; Carmona-Halty et al., 2019), and Chinese validation work has also reported acceptable psychometric properties (Fang et al., 2008; Meng, 2017). Although previous studies have linked sleep problems and mental health difficulties with weaker learning adaptation and academic engagement (Barbayannis et al., 2022; Ng et al., 2022; Sinval et al., 2025), less is known about the proximal cognitive process through which daytime sleepiness is connected with engagement in everyday study contexts.

Mind wandering provides a theoretically relevant proximal cognitive process. It refers to a shift of attention away from the current task toward task-unrelated internal thoughts. Educational research has shown that mind wandering is associated with poorer online learning experience and weaker learning outcomes (Wong et al., 2023; Wong et al., 2022). These findings justify examining whether mind wandering statistically accounts for part of the daytime sleepiness-academic engagement association. In the present cross-sectional design, however, this interpretation remains statistical and does not establish an attention mechanism or temporal sequence.

**Figure 1:**
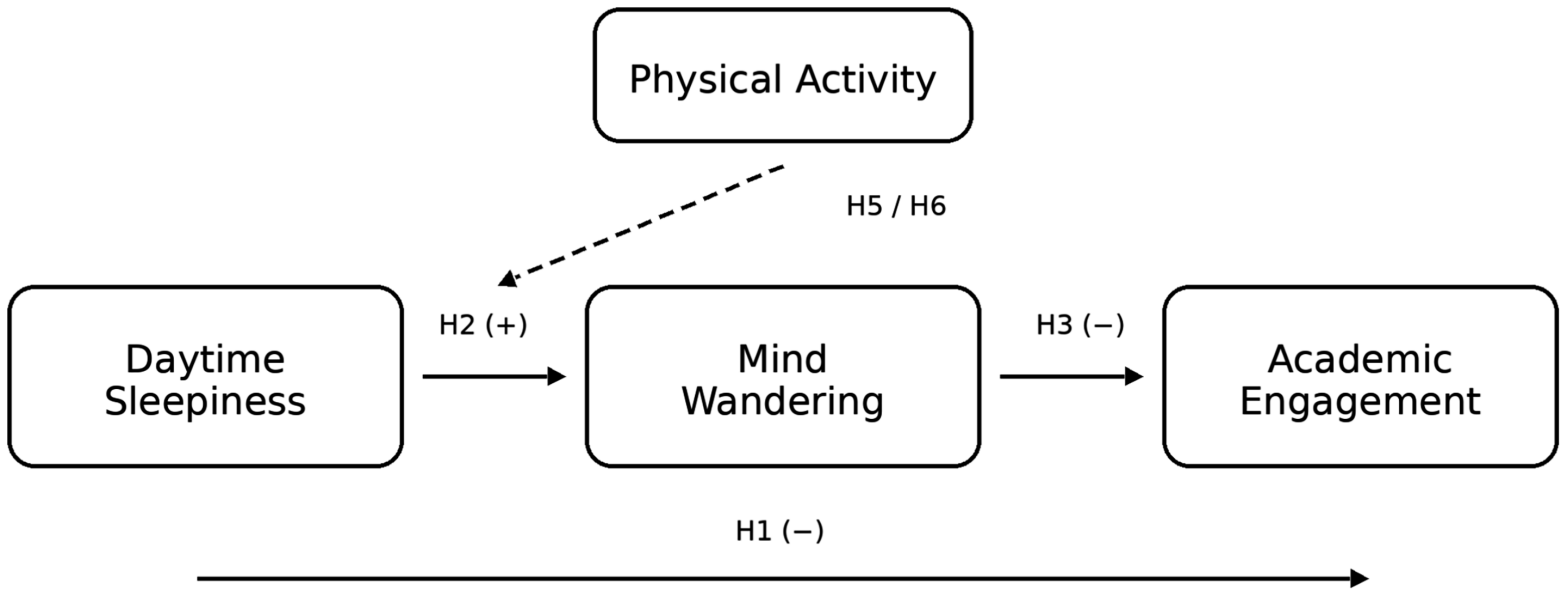
Hypothesized conceptual model. Solid arrows indicate the hypothesized direct and indirect paths; the dashed arrow indicates the moderating role of physical activity on the path from daytime sleepiness to mind wandering. For brevity, covariates (gender, grade, BMI, nighttime sleep duration, and nap frequency) are not shown in the figure but were included in all regression models.

**Figure 2:**
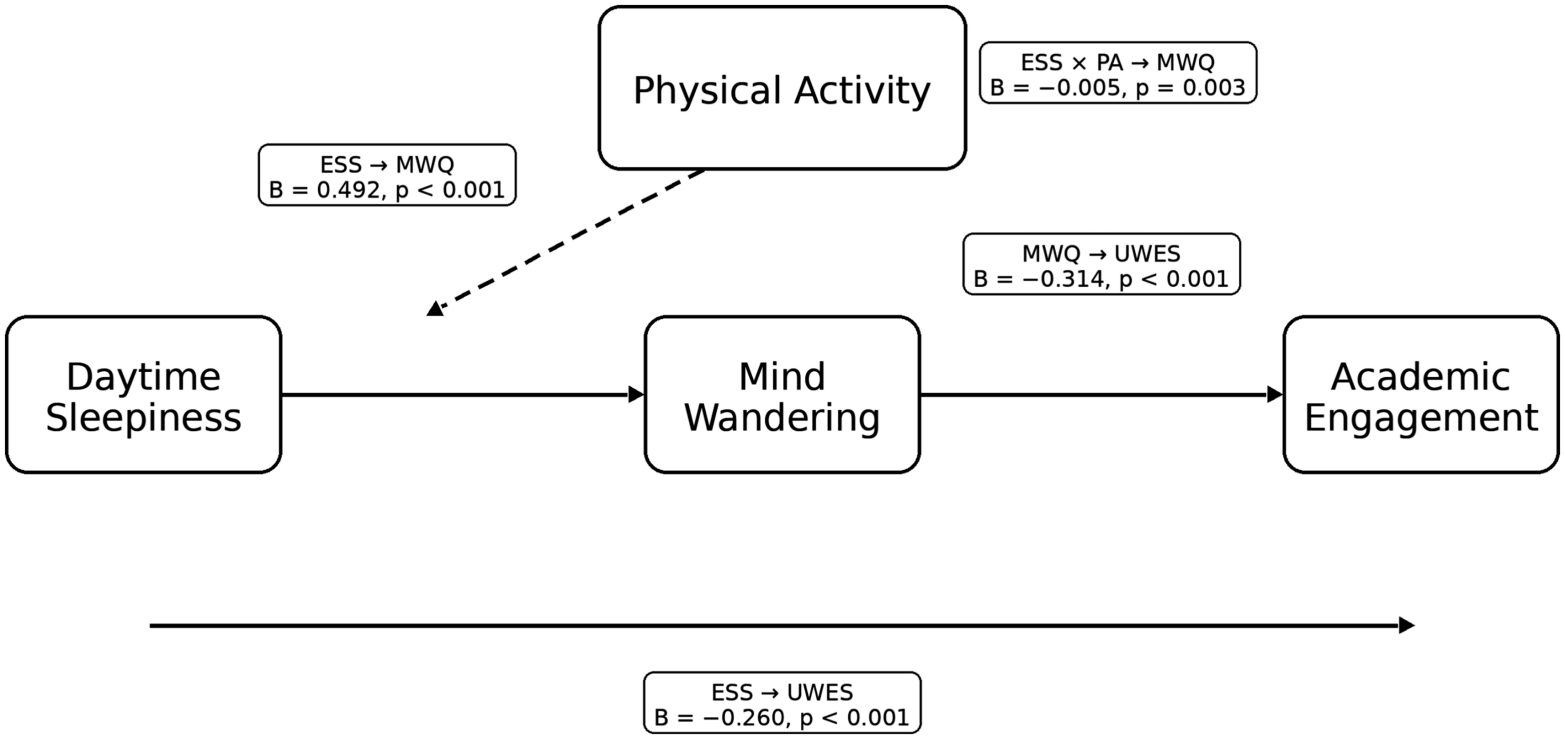
Moderated mediation model with unstandardized coefficients from the complete-case analysis (*n* = 1,371). Covariates were included in all models but are omitted from the figure for clarity.

**Figure 3:**
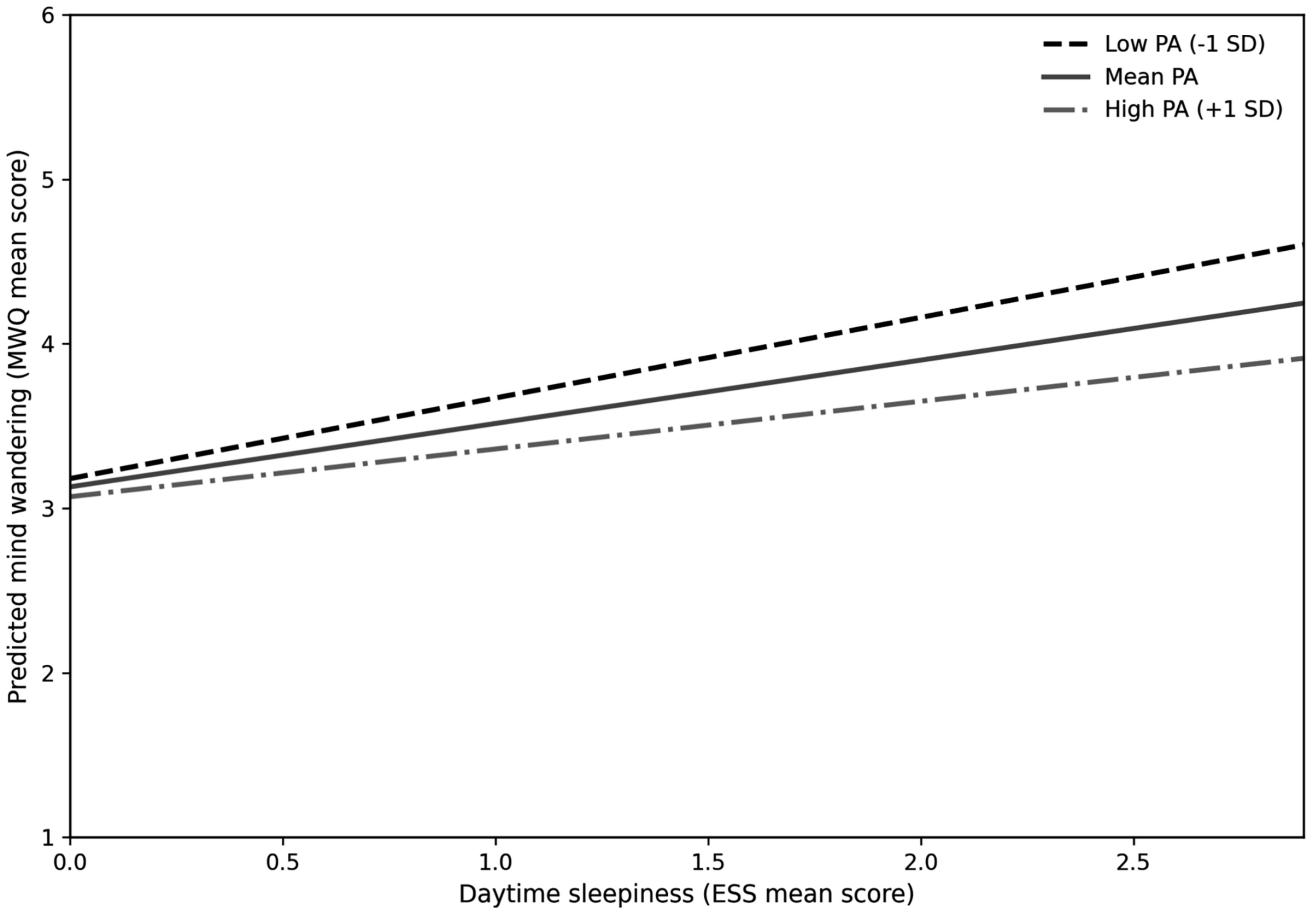
Conditional association between daytime sleepiness and mind wandering at low, mean, and high levels of physical activity. Slopes are shown on the full outcome scale; the near-overlap of the three lines reflects the very small interaction effect size.

Physical activity may also represent a candidate boundary condition in this association. Daily-life evidence indicates that physical activity, affect, sleep, and mind wandering are interrelated (Fanning et al., 2016), and brief exercise breaks may improve on-task attention during learning (Fenesi et al., 2018). Reviews and meta-analytic evidence further suggest that physical activity is associated with better mental health and sleep-related functioning (Rebar et al., 2015; Mikkelsen et al., 2017; Ghrouz et al., 2019; Mahindru et al., 2023; Ruiz-Ranz and Asin-Izquierdo, 2025). These findings support testing physical activity as a possible moderator, but they do not imply a strong protective effect. Therefore, the present study evaluated not only whether moderation was statistically detectable but also whether its magnitude was practically meaningful (Figures 1–3).

The present study therefore addressed a specific gap in the literature: previous research has examined sleepiness, mind wandering, engagement, and physical activity separately, but fewer studies have integrated them into a single conditional process model in university students. We tested six hypotheses using cross-sectional data: H1, daytime sleepiness would be negatively associated with academic engagement; H2, daytime sleepiness would be positively associated with mind wandering; H3, mind wandering would be negatively associated with academic engagement; H4, mind wandering would statistically mediate the association between daytime sleepiness and academic engagement; H5, physical activity would moderate the association between daytime sleepiness and mind wandering; and H6, physical activity would further moderate the indirect association between daytime sleepiness and academic engagement through mind wandering. All hypotheses were interpreted as associational rather than causal because of the cross-sectional design.

## 2 Materials and methods

### 2.1 Participants and procedure

A convenience sample of students from universities in Guizhou Province, China, completed an anonymous online self-administered questionnaire. The survey link was distributed through university-related student networks, and participation was voluntary. Before accessing the questionnaire items, participants were informed of the study purpose, anonymity, confidentiality, approximate completion time, and their right to withdraw at any point. All participants provided electronic informed consent before completing the questionnaire. No financial or course-credit incentives were provided. The questionnaire included demographic items, daytime sleepiness, mind wandering, an attention-check item, academic engagement, and physical activity. A total of 1,466 questionnaires were initially collected. After the predefined data-quality rules had been applied, 1,377 valid cases were retained for the main analyses. Raw-data inspection indicated that all retained cases passed the attention-check item and exceeded the minimum response-time criterion of 60 s; among excluded cases, failed attention checks were universal and 21 additionally fell below 60 s. In analyses involving physical activity, 6 additional cases had missing values on the relevant analysis items and were therefore excluded, resulting in a complete-case subsample of 1,371. The study was reviewed and approved by the Ethics Committee of Xinjiang Normal University (Approval No. XJNU2026LLSC50), and all procedures complied with the Declaration of Helsinki.

### 2.2 Measures

All psychometric instruments were administered in Chinese. When available, previously validated Chinese versions were used; otherwise, established original instruments were used with wording checked by the research team for semantic clarity in the student survey context.

#### 2.2.1 Epworth Sleepiness Scale

Daytime sleepiness was measured with the Epworth Sleepiness Scale (ESS), which assesses the likelihood of dozing in eight common daily situations (Johns, 1991). The Chinese version of the ESS has shown acceptable reliability and validity in prior validation work (Chen et al., 2002). Each item is scored from 0 to 3, with higher scores indicating greater daytime sleepiness. In the present study, the mean item score (ESS_mean) was used in the analyses.

#### 2.2.2 Mind-Wandering Questionnaire

Mind wandering was measured with the Mind-Wandering Questionnaire (MWQ), a five-item measure of task-unrelated thought in everyday activities (Mrazek et al., 2013). A Chinese version of the MWQ has demonstrated acceptable reliability and validity among Chinese university students (Ju et al., 2016). Items are rated on a 1–6 scale, and higher scores indicate more frequent mind wandering. The mean item score (MWQ_mean) was used.

#### 2.2.3 Utrecht Work Engagement Scale-Student version

Academic engagement was measured with the Chinese student version of the Utrecht Work Engagement Scale (UWES-S-9), which assesses vigor, dedication, and absorption in study activities (Fang et al., 2008; Meng, 2017). The scale contains nine items rated from 0 to 6, with higher scores indicating greater academic engagement. The mean item score (UWES_mean) was entered into the models.

#### 2.2.4 Physical Activity Rating Scale-3

Physical activity was measured with the Physical Activity Rating Scale-3 (PARS-3), a Chinese instrument commonly used to assess exercise intensity, duration, and frequency in student samples (Liang, 1994). The physical activity score was calculated as intensity × (duration–1) × frequency, yielding a score range from 0 to 100. Higher scores indicate greater physical activity. In the present study, the continuous PARS_total score was used as the moderator.

#### 2.2.5 Covariates

Gender, grade, body mass index (BMI), average nighttime sleep duration, and nap frequency were included as covariates in all regression models. These covariates were selected because demographic characteristics and general sleep behaviors may be related to both daytime sleepiness and academic engagement.

### 2.3 Statistical analysis

An *a priori* power analysis was conducted for linear multiple regression. Using a small effect size (f^2^ = 0.02), alpha = 0.05, powe*r* = 0.80, one focal predictor for the incremental test, and eight predictors in the most complex model, the required sample size was approximately 395. The final complete-case sample used in the moderated mediation analysis (*n* = 1,371) exceeded this requirement, indicating adequate statistical power to detect small effects.

All analyses were conducted in SPSSAU and were supplemented by raw-data checks. Skewness and kurtosis were examined to evaluate distributional characteristics, with absolute skewness values below 2 and absolute kurtosis values below 7 considered acceptable for approximate normality (Kim, 2013). Scatterplots and residual plots were inspected to assess linearity and homoscedasticity. Variance inflation factors were examined to evaluate multicollinearity, and no severe multicollinearity was detected; the maximum VIF in the moderated model was 4.56. Pearson correlations were then computed for the main study variables.

Common method bias was evaluated with Harman’s single-factor test as a preliminary diagnostic procedure (Podsakoff et al., 2003). The mediation model was tested with bootstrap procedures for indirect effects (Preacher and Hayes, 2008), implemented in Hayes’ PROCESS framework (Hayes, 2013). The moderated mediation model was estimated using conditional process analysis (Hayes and Rockwood, 2020), with the broader logic of integrating moderation and mediation following established methodological guidance (Edwards and Lambert, 2007). All indirect effects and the index of moderated mediation were evaluated with 5,000 bootstrap resamples and 95% confidence intervals. Because the moderated mediation model required complete data on physical activity, slight coefficient differences between the mediation and moderated mediation models were expected and reflect the difference between the *n* = 1,377 and *n* = 1,371 analytic samples.

## 3 Results

### 3.1 Common method bias

All 25 items from the ESS, MWQ, UWES-S-9, and PARS-3 were entered into an exploratory factor analysis as a preliminary diagnostic check for common method variance. The Kaiser-Meyer-Olkin value was 0.922 and Bartlett’s test of sphericity was significant (*p* < 0.001). Four factors with eigenvalues greater than 1 were extracted, and the first unrotated factor accounted for 25.836% of the variance, which is below the conventional 50% threshold. This result indicates that no single factor dominated the item variance. However, because this assessment still relies on Harman’s single-factor test, it should not be interpreted as evidence that common method bias was absent. Residual common method variance remains possible and is explicitly addressed in the limitations.

### 3.2 Sample characteristics, reliability, and descriptive statistics

Sample characteristics are summarized in Table 1. Reliability coefficients and descriptive statistics for the main variables are shown in Table 2. Cronbach’s alpha values were 0.873 for the ESS, 0.800 for the MWQ, 0.841 for the UWES-S-9, and 0.758 for the PARS-3, indicating acceptable to good internal consistency. No participant reached the theoretical maximum ESS mean score of 3.0; the observed maximum was 2.875.

**Table 1:** Sample characteristics (*n* = 1,377).

Variable	Category	n	%
Gender	Male	652	47.35
	Female	725	52.65
Grade	First-year undergraduate	345	25.05
	Second-year undergraduate	370	26.87
	Third-year undergraduate	321	23.31
	Fourth-year undergraduate	198	14.38
	Postgraduate	143	10.38
Nap frequency	Almost never	259	18.81
	1–2 times/week	392	28.47
	3–5 times/week	434	31.52
	Almost every day	292	21.21
Age (years)	Mean ± SD	20.64 ± 1.59	-

Age is reported as mean ± SD; all other values are frequencies and percentages.

**Table 2:** Reliability and descriptive statistics of the main variables.

Variable	Items	*n*	Cronbach α	Range	Mean ± SD	Median
ESS	8	1,377	0.873	0.000–2.875	1.000 ± 0.630	1.000
MWQ	5	1,377	0.800	1.200–6.000	3.507 ± 0.817	3.500
UWES	9	1,377	0.841	1.444–5.667	3.503 ± 0.748	3.444
PARS	3	1,371	0.758	0.000–100.000	21.259 ± 20.851	16.000
BMI	1	1,377	-	15.389–34.415	21.560 ± 3.160	21.304
Nighttime sleep duration (h)	1	1,377	-	4.000–10.000	6.987 ± 1.064	7.000

ESS, Epworth Sleepiness Scale; MWQ, Mind-Wandering Questionnaire; UWES, Utrecht Work Engagement Scale; PARS = Physical Activity Rating Scale; BMI = body mass index. PARS total score was calculated as intensity × (duration–1) × frequency. Analyses involving physical activity were based on *n* = 1,371 complete cases; all other variables were based on *n* = 1,377. The maximum observed ESS mean score was 2.875, indicating that no participant endorsed the maximum possible score across all eight items.

### 3.3 Correlation analysis

Pearson correlations are shown in Table 3. Daytime sleepiness was positively associated with mind wandering (*r* = 0.334, *p* < 0.001) and negatively associated with academic engagement (*r* = −0.334, *p* < 0.001). Mind wandering was negatively associated with academic engagement (*r* = −0.414, *p* < 0.001). Physical activity was negatively associated with mind wandering (*r* = −0.206, *p* < 0.001) and positively associated with academic engagement (*r* = 0.240, *p* < 0.001). Nighttime sleep duration was negatively associated with daytime sleepiness (*r* = −0.359, *p* < 0.001) and positively associated with academic engagement (*r* = 0.147, *p* < 0.001). Overall, the correlation coefficients were small to moderate in magnitude, indicating statistically detectable but not deterministic associations among the study variables.

**Table 3:** Pearson correlations among the main variables.

Variable	M	SD	1	2	3	4	5	6	7
1 ESS	1.000	0.630	1						
2 MWQ	3.507	0.817	0.334***	1					
3 UWES	3.503	0.748	−0.334***	−0.414***	1				
4 PARS	21.259	20.851	0.006	−0.206***	0.240***	1			
5 BMI	21.560	3.160	0.005	0.002	−0.023	0.024	1		
6 Sleep duration	6.987	1.064	−0.359***	−0.196***	0.147***	0.013	0.013	1	
7 Nap frequency	2.551	1.024	0.023	0.031	0.007	−0.029	0.001	−0.018	1

ESS, Epworth Sleepiness Scale; MWQ, Mind-Wandering Questionnaire; UWES, Utrecht Work Engagement Scale; PARS, Physical Activity Rating Scale; BMI, body mass index. ***p < 0.001. Correlations involving PARS were based on *n* = 1,371; the remaining correlations were based on *n* = 1,377. Coefficients are rounded to three decimals; the apparent equality in absolute value for the ESS-MWQ and ESS-UWES correlations reflects rounding of the raw-data estimates (0.3335 and −0.3338).

### 3.4 Mediation analysis

The mediation hypotheses (H1-H4) were tested first. As shown in Table 4, daytime sleepiness was positively associated with mind wandering in the regression model [*a* = 0.391, 95% CI (0.322, 0.460), *p* < 0.001], and mind wandering was negatively associated with academic engagement [*b* = −0.312, 95% CI (−0.358, −0.266), *p* < 0.001]. The total association between daytime sleepiness and academic engagement was significant [*c* = −0.384, 95% CI (−0.447, −0.320), *p* < 0.001]. After mind wandering was entered into the model, the direct association remained significant but was attenuated [*c*’ = −0.262, 95% CI (−0.324, −0.200), *p* < 0.001]. The bootstrap indirect effect was significant [*B* = −0.122, 95% bootstrap CI (−0.152, −0.094)], indicating partial statistical mediation. The indirect effect accounted for approximately 31.8% of the total association, suggesting a nontrivial but not complete explanatory pathway.

**Table 4:** Key coefficients from the mediation model.

Path	Symbol	B	95% CI lower	95% CI upper	SE	*p*	Interpretation
ESS - > MWQ	a	0.391	0.322	0.460	0.035	<0.001	Positive
MWQ - > UWES	b	−0.312	−0.358	−0.266	0.023	<0.001	Negative
ESS - > UWES (total effect)	c	−0.384	−0.447	−0.320	0.032	<0.001	Significant
ESS - > UWES (direct effect)	c’	−0.262	−0.324	−0.200	0.032	<0.001	Significant
ESS - > MWQ - > UWES	a*b	−0.122	−0.152	−0.094	0.015	<0.001	Partial mediation

ESS, Epworth Sleepiness Scale; MWQ, Mind-Wandering Questionnaire; UWES, Utrecht Work Engagement Scale; BMI, body mass index; CI, confidence interval; SE, standard error. Unstandardized B coefficients are reported. Covariates included gender, grade, BMI, nighttime sleep duration, and nap frequency. The indirect effect accounted for approximately 31.8% of the total association.

### 3.5 Moderated mediation analysis

The moderated mediation hypotheses (H5-H6) were then tested. The moderated mediation results are presented in Tables 5, 6. In the model with mind wandering as the outcome, daytime sleepiness was positively associated with mind wandering (*B* = 0.492, *p* < 0.001), the main effect of physical activity was marginal (*B* = −0.003, *p* = 0.058), and the interaction between daytime sleepiness and physical activity was statistically significant (*B* = −0.005, *p* = 0.003). Adding the interaction term increased explained variance by only ΔR^2^ = 0.005, indicating a small incremental contribution. In the model with academic engagement as the outcome, mind wandering was negatively associated with academic engagement (*B* = −0.314, *p* < 0.001), and the direct association involving daytime sleepiness remained significant (*B* = −0.260, *p* < 0.001). The model explained 16.9% of the variance in mind wandering and 21.9% of the variance in academic engagement.

**Table 5:** Regression coefficients from the moderated mediation model.

Outcome	Predictor	B	SE	t	p	R^2^
MWQ	ESS	0.492	0.047	10.536	<0.001	0.169
MWQ	PARS	−0.003	0.002	−1.899	0.058	
MWQ	ESS x PARS	−0.005	0.002	−3.000	0.003	
UWES	ESS	−0.260	0.032	−8.152	<0.001	0.219
UWES	MWQ	−0.314	0.023	−13.416	<0.001	

ESS, Epworth Sleepiness Scale; MWQ, Mind-Wandering Questionnaire; UWES, Utrecht Work Engagement Scale; PARS, Physical Activity Rating Scale; BMI, body mass index; SE, standard error; R^2^, variance explained; ΔR^2^, change in explained variance. Unstandardized B coefficients are reported. The moderated mediation model was estimated on the complete-case subsample with valid physical-activity data (*n* = 1,371). Covariates were included in both regression models but are not displayed in the table. For the MWQ model, adding the ESS × PARS interaction increased explained variance by ΔR^2^ = 0.005 (*p* = 0.003), indicating a small incremental contribution.

**Table 6:** Conditional indirect effects at different levels of physical activity.

Physical activity level	Value	Indirect effect	Boot SE	95% CI lower	95% CI upper	Interpretation
Low (−1 SD)	0.408	−0.154	0.019	−0.192	−0.119	Significant
Mean	21.259	−0.124	0.015	−0.155	−0.097	Significant
High (+1 SD)	42.110	−0.095	0.018	−0.130	−0.062	Significant
Index of moderated mediation	-	0.001	0.001	0.000	0.002	Very small; interpret cautiously

PARS, physical activity rating scale; CI, confidence interval; Boot SE, bootstrap standard error. Low, mean, and high levels of physical activity correspond to the mean minus one standard deviation, the mean, and the mean plus one standard deviation, respectively. The final line reports the index of moderated mediation and its 95% bootstrap confidence interval.

Conditional indirect effects were negative at low (−1 SD), mean, and high (+1 SD) levels of physical activity, and their absolute magnitudes decreased numerically as physical activity increased. However, the moderated mediation index was extremely small (index = 0.001, 95% bootstrap CI [0.000, 0.002]), and the interaction added only a small amount of explained variance. Thus, although the moderation was statistically detectable, its practical relevance appears limited. This result should not be interpreted as evidence that physical activity meaningfully or strongly buffers the association between daytime sleepiness and mind wandering.

## 4 Discussion

### 4.1 Principal findings

This study examined an associational conditional process model linking daytime sleepiness, mind wandering, academic engagement, and physical activity among university students. The results supported H1-H4: daytime sleepiness was associated with lower academic engagement, daytime sleepiness was associated with more frequent mind wandering, mind wandering was associated with lower academic engagement, and mind wandering partially accounted for the sleepiness-engagement association. H5 and H6 received only limited support: physical activity was involved in a statistically detectable interaction, but the effect size was very small.

### 4.2 Direct association between daytime sleepiness and academic engagement

Consistent with H1, students reporting higher daytime sleepiness also reported lower academic engagement after adjustment for gender, grade, BMI, nighttime sleep duration, and nap frequency. This finding aligns with prior work linking excessive daytime sleepiness and poor sleep quality with weaker academic functioning. Importantly, the present result should be interpreted as an association rather than evidence that sleepiness causes disengagement. Daytime sleepiness may operate alongside other unmeasured factors, including sleep quality, chronotype, mood symptoms, caffeine or stimulant use, and ADHD-related symptoms.

### 4.3 Mind wandering as a partial statistical mediator

The results for H2-H4 suggest that mind wandering is a proximal cognitive correlate of the association between daytime sleepiness and academic engagement. The indirect effect accounted for approximately 31.8% of the total association, indicating that task-unrelated thought was involved in a meaningful part of the statistical link. At the same time, the direct association remained significant, suggesting partial rather than complete mediation. This pattern is consistent with attention-resource accounts, but the present study did not directly measure cognitive control, alertness physiology, or temporal change. Therefore, the mediation model should be understood as a theoretically informed statistical model rather than evidence of a temporal or causal psychological mechanism.

### 4.4 Physical activity as a statistically detectable but limited moderator

The findings for H5-H6 require a more conservative interpretation. The interaction between daytime sleepiness and physical activity was statistically significant, and the conditional indirect effects decreased numerically from low to high physical activity levels. However, the interaction added only a very small amount of explained variance (ΔR^2^ = 0.005), and the index of moderated mediation was 0.001 with a confidence interval very close to zero. These values indicate limited practical relevance. Accordingly, physical activity should not be described as a strong or clinically meaningful buffering factor in this model. The most cautious interpretation is that physical activity was associated with a slightly weaker daytime sleepiness-mind wandering association, but the magnitude of this moderation was small.

### 4.5 Theoretical and practical implications

Theoretically, the study contributes to educational psychology by examining daytime sleepiness, mind wandering, and academic engagement within a single associational model. The findings provide stronger support for the mind-wandering component than for the physical activity boundary condition. Practically, the results should be used cautiously. They do not justify treating general self-reported physical activity as a stand-alone solution for sleepiness-related academic difficulties. Instead, they suggest that student support efforts may need to focus primarily on sleep-related education, attention support, and behavioral schedule management, while physical activity should be considered only as a potentially useful but limited complementary factor. Because this study did not test an intervention, specific intervention effects should be examined in future longitudinal or experimental studies.

### 4.6 Limitations and future directions

Several limitations should be noted. First, the cross-sectional design prevents conclusions about temporal order or causality; longitudinal, time-lagged, ecological momentary assessment, or experimental designs are needed to clarify directionality. Second, all focal variables were collected through self-report questionnaires at the same time point. Although Harman’s single-factor test did not indicate a dominant single factor, this test is only a preliminary diagnostic procedure and cannot rule out common method variance. Because the assessment of common method bias in this study still depended mainly on this procedure, residual common method bias should be explicitly acknowledged. Future studies should incorporate multi-source data, objective sleep or activity indicators, wearable-device measures, marker variables, or time-separated assessments. Third, residual confounding remains possible because anxiety symptoms, depressive symptoms, ADHD-related symptoms, sleep quality, sleep fragmentation, chronotype, and caffeine or stimulant use were not assessed. Fourth, PARS-3 captures general physical activity volume but does not distinguish activity type, timing, weekday/weekend patterns, or acute post-exercise alertness. Fifth, the sample was drawn from universities in one Chinese province, which limits generalizability to other regions and institutional contexts. Finally, although the sample size was adequate to detect small effects, the statistically significant moderation may partly reflect the large sample and should be interpreted in terms of effect size rather than *p* value alone.

## 5 Conclusion

In this cross-sectional study, daytime sleepiness was associated with lower academic engagement among university students after adjustment for gender, grade, BMI, nighttime sleep duration, and nap frequency. Mind wandering partially accounted for this association, suggesting that task-unrelated thought is a relevant proximal cognitive correlate. Physical activity showed a statistically detectable but very small moderating association with the daytime sleepiness-mind wandering path. Overall, the findings support a cautious associational interpretation: lower daytime sleepiness and less frequent mind wandering were linked to higher academic engagement, whereas the moderation by physical activity had limited practical relevance and should not be overstated.

## Data Availability

The datasets presented in this article are not readily available because the raw anonymized data supporting the conclusions of this article are available from the corresponding author upon reasonable request. The data are not publicly deposited because they contain participant-level survey responses and are subject to privacy and institutional ethics considerations. Requests to access the datasets should be directed to the corresponding author.
